# Advancing cancer treatment: *in vivo* delivery of therapeutic small noncoding RNAs

**DOI:** 10.3389/fmolb.2023.1297413

**Published:** 2024-01-03

**Authors:** Xiaoyue Yang, Ying Liang, Sheng Tong

**Affiliations:** ^1^ F. Joseph Halcomb III, MD Department of Biomedical Engineering, University of Kentucky, Lexington, KY, United States; ^2^ New York Blood Center, New York, NY, United States

**Keywords:** cancer, small non-coding RNA, miRNA, siRNA, *in vivo* delivery

## Abstract

In recent years, small non-coding RNAs (ncRNAs) have emerged as a new player in the realm of cancer therapeutics. Their unique capacity to directly modulate genetic networks and target oncogenes positions them as valuable complements to existing small-molecule drugs. Concurrently, the advancement of small ncRNA-based therapeutics has rekindled the pursuit of efficacious *in vivo* delivery strategies. In this review, we provide an overview of the most current clinical and preclinical studies in the field of small ncRNA-based cancer therapeutics. Furthermore, we shed light on the pivotal challenges hindering the successful translation of these promising therapies into clinical practice, with a specific focus on delivery methods, aiming to stimulate innovative approaches to address this foundational aspect of cancer treatment.

## 1 Introduction

Non-coding RNAs (ncRNAs), comprising RNA molecules that do not undergo translation into proteins, represent over 90% of total RNA transcripts. These ncRNAs can be categorized based on their length as long ncRNAs (>200 nucleotides) and small ncRNAs (18–200 nucleotides). Small ncRNAs include small interfering RNA (siRNA), microRNA (miRNA), small PIWI-interacting RNA (piRNA), and transfer RNA (tRNA)-derived small RNAs (Zhang et al., 2021). Since the discovery of RNA interference (RNAi) in the 1990s, our perception of ncRNA has evolved from viewing them as mere byproducts to recognizing their pivotal roles as the regulators of genetic networks (Friedman et al., 2009). Consequently, small ncRNAs have emerged as a significant class of therapeutics for various diseases. The pursuit of small ncRNA therapeutics reached a milestone with the FDA approval of Patisiran in 2018, marking the first siRNA drug for treating hereditary transthyretin amyloidosis (hATTR). Today, many small ncRNA therapeutics for cancer treatment are undergoing clinical trials, holding the promise of breakthrough therapies in the near future.

Depending on their intracellular localization and interactions with DNAs, RNAs, or proteins, ncRNAs play a pivotal role in cancer development and progression by modulating transcription, RNA splicing, or translation processes (Anastasiadou et al., 2018; Statello et al., 2021). Notably, dysregulation of miRNAs often contributes to aberrant activities of both oncogenes and tumor suppressor genes (Calin and Croce, 2006; Esquela-Kerscher and Slack, 2006). For instance, in various cancer types, including pancreatic cancer and lung cancer where *KRAS* mutations are key oncogenic drivers (Buscail et al., 2020), several miRNAs (*miR-143*, *miR-145*, *miR-216*, *miR-217*, and *let-7*) binding to the 3′ untranslated region (3′UTR) of *KRAS* mRNA are downregulated, thereby contributing to the hyperactivity of KRAS mutants (Volinia et al., 2006; Szafranska et al., 2007). Conversely, oncogenic miRNAs (*let-1b*, *miR-21*, *miR-135*, *miR-141*, and *miR-205*) that target tumor-suppressive genes, such as *PTEN*, are often upregulated, inhibiting the tumor suppressor functions (Chen et al., 2019; Vahabi et al., 2021). Moreover, certain miRNAs like *miR-21* and *miR-27a* have been implicated in conferring resistance to radiotherapy and chemotherapy (Li et al., 2010; Sheng et al., 2022). In this context, ncRNAs offer significant opportunities for therapeutic interventions in cancer treatment.

**FIGURE 1:**
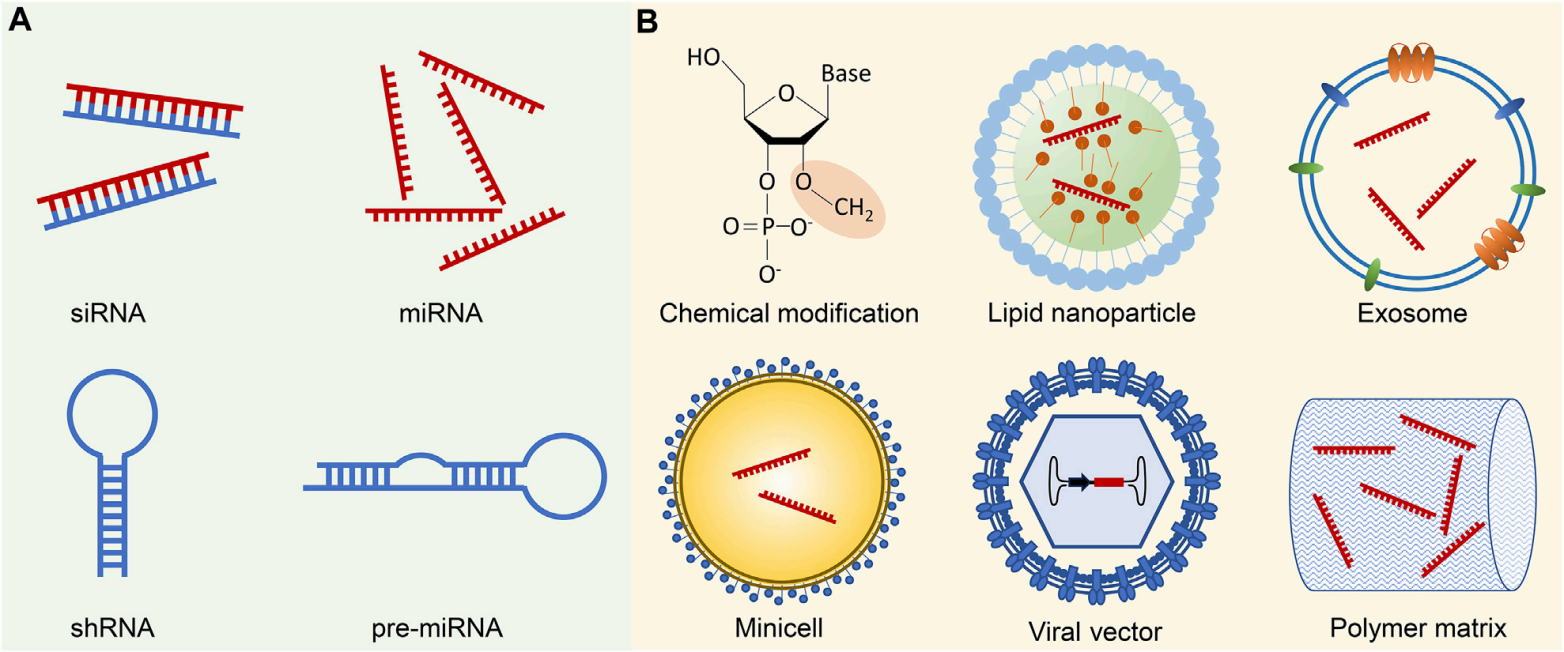
Small ncRNA therapeutics and delivery methods. **(A)** Small ncRNA-based therapeutics primarily involve siRNAs and miRNAs, which can be administered either in their mature forms or as precursor molecules, such as shRNAs and pre-miRNAs. While shRNAs and pre-miRNAs can be generated using plasmids or viral vectors, their transformation into mature forms necessitates additional processing in the cells. **(B)** Successful delivery of small ncRNA therapeutics is challenged by several biological barriers, including enzymatic degradation, immune system activation, endosomal entrapment, and off-target effects. Chemical modification of ncRNA backbones, such as 2′-O-Me modification of 2′-ribose hydroxyl sites, contributes to increased stability, reduced immunogenicity, and enhanced binding affinity to the target sequence. To facilitate targeted delivery, small ncRNAs are often encapsulated in various delivery vehicles. Common examples include synthetic nanoparticles, such as lipid nanoparticles, or biogenic nano and macro-vesicles like exosomes or minicells. Viral vectors, with the ncRNA expression cassette inserted into the viral genome, represent an alternative delivery approach. Moreover, ncRNAs can be incorporated into polymer pellets for sustained local release.

To date, the development of small ncRNA therapeutics has predominantly revolved around two extensively studied classes: siRNA and miRNA (Figure 1A). Small ncRNA therapeutics possess several distinct advantages over conventional small-molecule drugs. Firstly, small ncRNAs interact with their targets through Watson-Crick base pairing, enabling rational design for any gene of interest. This feature holds particular importance for therapeutic targets that are deemed “undruggable” by small-molecular drugs, such as the *KRAS* ^*G12D*^ mutant. All small ncRNAs share similar physical and chemical properties, resulting in analogous pharmacokinetics in the body. Consequently, a delivery method proven effective for one small ncRNA is likely to apply to others. Additionally, each miRNA can concurrently target multiple genes, potentially amplifying therapeutic effects by regulating several nodes in a pathologic pathway (Goodall and Wickramasinghe, 2021). However, small ncRNAs also pose new challenges for therapeutic applications, including issues related to stability, immunogenicity, off-target effects, and limited on-target efficiency (Kulkarni et al., 2019). Over the past decade, substantial progress has been made in developing chemical modifications to enhance the stability and reduce the immunogenicity of RNAs. Nevertheless, the efficient delivery of small ncRNAs remains a major obstacle in extending their utility beyond the liver. This article discusses the challenges associated with *in vivo* delivery of small ncRNAs and provides an overview of current clinical trials and the latest advancement in the field, to inspire the creation of novel delivery platforms for safe and effective small ncRNA therapeutics.

## 2 Challenges in small ncRNA therapeutics

Small ncRNA-based cancer therapeutics include siRNAs, miRNA mimics, and miRNA inhibitors (antimiRs). Although both siRNAs and miRNAs operate in the RNAi pathway, siRNAs have achieved more clinical success, likely due to their superior specificity. Recent advancements in bioinformatics and sequencing technologies have provided many tools for high-throughput screening of small ncRNA candidates. In addition to target selection, effective cancer treatment requires the efficient delivery of small ncRNA therapeutics to their molecular targets, typically cytoplasmic mRNAs in the cancer cells, while minimizing unintentional on-target effects in normal tissues. RNA molecules, due to their negative charge and hydrophilicity, cannot cross the cell membrane. *In vitro* applications allow for the delivery of RNA molecules into the cytoplasm through lipid transfection or electroporation. However, *in vivo* applications face multiple transport barriers within the systemic circulation and the tumor tissue.

Natural RNA molecules are highly susceptible to enzymatic degradation by serum and cellular RNases. Notably, both single-stranded and double-stranded RNAs can trigger the body’s viral defense system via pathogen-associated molecular patter (PAMP) receptors (Kumar et al., 2011), a phenomenon led to the termination of several clinical trials (Kleinman et al., 2008). Since then, various chemical modifications have been developed to enhance RNA stability and reduce immunogenicity. These modifications include replacing phosphodiester with phosphorothioate, modifying 2′-ribose hydroxyl site with 2′-F, 2′-*O*-Me, or 2′-MOE, and introducing structural modifications like lock nucleic acids (LNAs), peptide nucleic acids (PNAs), and phosphoramidate morpholino oligomers (PMOs) (Winkle et al., 2021). The structural modifications can also increase binding affinity and improve the selection of the guide strand in double-stranded ncRNAs.

Much like small-molecule drugs, small ncRNA therapeutics can be administrated systemically or locally. Systemic administration necessitates the distribution of small ncRNAs through the circulatory system, extravasation from blood vessels, and movement through the interstitial space to reach the target cells. The circulation half-life of unmodified small RNAs is only a few minutes due to rapid enzymatic degradation and renal clearance (Gao et al., 2009; Iversen et al., 2013). With few exceptions, small ncRNA therapeutics are formulated with synthetic or biogenic nanoparticles, which improve stability, circulation time, and tumor targeting. However, loading into nanoparticles significantly alters the pharmacokinetics of small ncRNAs and subsequently, their distribution in the body (Jain and Stylianopoulos, 2010). Generally, circulating nanoparticles are primarily sequestrated by the mononuclear phagocytic system (MPS) in the liver, spleen, and lymph nodes, while those larger than 200 nm can be removed by splenic filtration (Blanco et al., 2015). Nanoparticles tend to accumulate in solid tumors due to the enhanced permeability and retention (EPR) effect. Nevertheless, nanoparticle extravasation from tumor vessels varies significantly due to the heterogenous vascular network in tumors and elevated interstitial pressure, which differs among tumor types and stages (Hobbs et al., 1998). Solid tumors also possess an extracellular matrix of excessive collagens and hyaluronic acids, a major barrier to interstitial transport (Yuan et al., 2001; Dreher et al., 2006). Moreover, most nanoparticles in tumor tissue are taken up by tumor-associated macrophages, diminishing their cargo’s availability to cancer cells (Dai et al., 2018). These transport barriers in tumors contribute to inaccessible niches, leading to tumor recurrence post-treatment.

Cell entry and endosomal escape represent additional barriers to delivering small ncRNA therapeutics. Both small ncRNAs and their nano formulations rely on endocytic pathways for cell entry. The cellular uptake can be significantly increased through receptor-mediated endocytosis. One important receptor for small ncRNA delivery is the asialoglycoprotein receptor (ASGPR) highly expressed by hepatocytes. Conjugation of its ligand, *N*-acetylgalactosamine (GalNAc), to siRNA substantially increases hepatocyte uptake (Shah and Giacca, 2022). GalNAc conjugated siRNAs present a unique case of gymnosis, i.e., siRNA delivery without excipients, thanks to their extremely high cellular uptake rate leading to opportunistic escape from endosomes. For most small ncRNAs, an endosomal release mechanism is necessary for reaching target mRNAs, often achieved by including excipients capable of destabilizing endosomal membranes, such as heptatriaconta-6,9,28,31-tetraen-19-yl-4-(dimethylamino) butanoate (DLin-MC3-DMA).

It is crucial to emphasize that addressing these delivery challenges demands a systematic approach. The compositions and configurations of the delivery vehicles can yield conflicting outcomes at different stages along the *in vivo* delivery route. For instance, compared to small ncRNAs, large nanoparticles face constraints in reaching cancer cells situated deeper within tissues due to their limited diffusivity and obstruction by the dense fibrous extracellular matrix. Moreover, nanoparticles designed to extend the circulation half-life of small ncRNAs may inadvertently reduce their cellular uptake by cancer cells. Consequently, the effectiveness of cancer treatment hinges on a meticulous balance of various facets of the delivery strategy, necessitating comprehensive *in vivo* evaluations tailored to specific disease conditions.

## 3 *In vivo* delivery methods in clinical and pre-clinical studies

To date, there have been over 30 clinical trials focusing on small ncRNA-based cancer therapeutics (Table 1), while numerous innovative formulations are currently under evaluation in preclinical studies. In this context, we aim to highlight a few recent breakthroughs that offer promising strategies for addressing the delivery obstacles associated with small ncRNA cancer therapeutics (Figure 1B).

**TABLE 1:** Clinical trials of small ncRNA cancer therapeutics.

Name	Formulation	Route of injection	Cancer type	Target gene	Identifier	Status
miRNA						
TargomiRs (miR-16 mimic)	Bacterially derived 400 nm particle	i.v.	MPM	BCL2, CCND1, CDK1, ETS1, JUN	NCT02369198	Phase I completed
MRX34 (miR-34a mimic)	Liposome	i.v.	Solid tumor	MET, MYC, PDGFR-α, CDK4/6, BCL2, PD-L1	NCT01829971	Phase I terminated
					NCT02862145	Phase I/II withdrawn
MRG-106 (miR-155 inhibitor)	LNA	i.v. & s.c.	MF-CTCL	JAK/STAT, PI3K/AKT, and MAPK pathways	NCT02580552	Phase I completed
					NCT03837457	Phase II terminated
					NCT03713320	Phase II terminated
siRNA						
Atu027	Liposome	i.v.	Solid tumors	PKN3	NCT00938574	Phase I completed
			Pancreatic cancer		NCT01808638	Phase I/II completed
siG12D LODER	PLGA matrix	Local release	LAPC	KRAS	NCT01188785	Phase I completed
					NCT01676259	Phase II
iExosome	MSC-derived Exosomes	i.v.	Pancreatic cancer	KRAS	NCT03608631	Phase I
TKM-080301	LNP	i.v.	NET and ACC	PLK1	NCT01262235	Phase I/II completed
			HCC		NCT02191878	Phase I/II completed
			Liver cancer		NCT01437007	Phase I completed
SXL01	Undisclosed	s.c.	Metastatic castration-resistant PCa	Androgen receptor	NCT02866916	Phase I withdrawn
EphA2 siRNA	DOPC neutral liposome	i.v.	Advanced or recurrent solid tumor	EphA2	NCT01591356	Phase I
NU-0129	Gold nanoparticle	i.v.	Glioblastoma	Bcl2L12	NCT03020017	Phase I completed
ALN-VSP02	Co-delivery of two siRNAs with LNP	i.v.	Advanced solid tumor with liver involvement	KSP and VEGF-A	NCT00882180	Phase I completed
			Responder in NCT00882180		NCT01158079	Phase I completed
CALAA-01	Cyclodextrin nanoparticles targeting transferrin receptor	i.v.	Solid tumor	Ribonucleotide reductase subunit M2	NCT00689065	Phase I terminated
DCR-MYC	LNP	i.v.	Solid tumor, multiple myeloma, or lymphoma	MYC	NCT02110563	Phase I terminated
			HCC		NCT02314052	Phase I/II terminated
CpG-STAT3	CpG conjugated siRNA	i.t.	r/r B-NHL	STAT3	NCT04995536	Phase I
STP705	Co-delivery of two siRNAs	i.t.	isSCC	TGF-β1 and COX-2	NCT04293679	Phase I completed
					NCT04844983	Phase II
			BCC		NCT04669808	Phase II
			CCA, HCC, or liver metastasis		NCT04676633	Phase I
PH-762	Cholesterol conjugated to the passenger strand	i.t.	cSCC, Melanoma, or MCC	PD-1	NCT06014086	Phase I
ARO-HIF2	Synthetic RNAi with α_v_β_3_ targeting ligand	i.v.	ccRCC	HIF-2α	NCT04169711	Phase I completed

Abbreviations. Cancer type: ACC, adrenocortical carcinoma; BCC, basal cell carcinoma; CCA, cholangiocarcinoma; ccRCC, clear cell renal cell carcinoma; cSCC, cutaneous squamous cell carcinoma; HCC, hepatocellular carcinoma; isSCC, squamous cell carcinoma *in situ*; LAPC, locally advanced pancreatic cancer; MCC, Merkel cell carcinoma; MF-CTCL, mycosis fungoides type cutaneous t-cell lymphoma. MPM, malignant pleural mesothelioma; NET, neuroendocrine tumor; NSCLC, non-small cell lung cancer; PCa, prostate cancer. r/r B-NHL, relapsed/refractory B-Cell non-Hodgkin lymphoma. Route of injection: i.v., intravenous injection. i.t., intratumoral injection. s.c., subcutaneous injection.

### 3.1 Local delivery

One key strategy for overcoming transport barriers involves the intratumoral injection of small ncRNAs, particularly in the context of locally advanced tumors. This localized approach mitigates the risks associated with eliciting immune responses and unintentional gene silencing in normal tissues. The primary objective here is to achieve optimal distribution and intracellular delivery of small ncRNAs within the primary tumor. Small RNAs, with appropriate chemical modifications, are often directly employed for injection, as their size significantly influences interstitial transport within the tumor. Furthermore, intratumoral distribution can be enhanced through convection-enhanced delivery. Another effective method involves cholesterol conjugation to the sense strand, facilitating intracellular delivery of siRNAs in the absence of transfection reagents (Soutschek et al., 2004). A related approach utilizes polymer implants to achieve sustained local release of siRNAs. For instance, a miniature biodegradable poly (lactic-co-glycolic) acid matrix (LODER) can release siRNAs over 2 months (Zorde Khvalevsky et al., 2013). In a Phase I clinical trial, patients with non-operable locally advanced pancreatic cancer (LAPC) received implantation of LODER containing the siRNA targeting the *KRAS* ^*G12D*^ mutant (NCT01188785) (Golan et al., 2015). This siRNA therapeutic was administrated alongside either Gemcitabine or FOLFIRINOX. Encouragingly, the study reported no dose-limiting toxicity, with 75% of patients displaying stable disease (SD) and 25% of patients exhibiting a partial response (PR) 4 months post-treatment. Subsequently, a phase II study (NCT01676259) was initiated to evaluate the therapeutic efficiency of LODER in combination with chemotherapy (Titze-de-Almeida et al., 2017; Varghese, et al., 2020).

### 3.2 Synthetic nanoparticles

Synthetic nanoparticles are employed for targeted delivery of small ncRNA therapeutics through systemic administration. Among various nano formulations, lipid nanoparticles (LNP) have garnered substantial recognition, partly owing to the FDA’s approval of Patisiran and more recently, COVID-19 mRNA vaccines. LNPs serve to safeguard ncRNAs against degradation, mitigate immune activation, enhance localization to the target tissue, and facilitate intracellular delivery (Adams et al., 2018; Kulkarni et al., 2019). A pivotal constituent in LNPs is ionizable cationic lipids that bind to siRNA. During the development of Patisiran, DLin-MC3-DMA with a pKa of approximately 6.4 was selected from hundreds of candidates following extensive screening. At a neutral pH, DLin-MC3-DMA maintains a neutral state, while within the acidic endosomes, it undergoes ionization, thereby merging with the endosomal membrane and enabling the release of RNAs into the cytoplasm. Prior studies have underscored that a deviation in pKa as slight as 0.5 units can substantially reduce therapeutic efficacy by over 100-fold. The optimal pKa highlights the necessity for LNPs to maintain a low surface charge to prevent clearance in circulation and then transition to a positive charge to facilitate endosomal escape. For cancer treatment, further refinement in structure and function is imperative due to the perturbed pH Homeostasis observed in the tumor microenvironment, stemming from metabolic alterations in cancer cells. Besides cationic lipids, LNPs can incorporate additional components such as cholesterol, fusogenic phospholipids, poly (ethylene glycol), and cancer cell-specific ligands to bolster structural stability, enhance transfection capabilities, evade immune surveillance, and increase tumor-targeting efficiency (Zatsepin et al., 2016; Adams et al., 2017). The intricate correlations among the composition, structure, and function of LNPs remain incompletely elucidated, necessitating the development of novel characterization techniques (Hammel et al., 2023).

### 3.3 Extracellular vesicles

Extracellular vesicles (EVs), including microvesicles and exosomes, are biogenic nanoparticles released by almost all cell types. EVs play crucial roles in a wide range of physiological and pathological processes by transferring bioactive contents, including microRNAs, among various cell populations (O'Driscoll, 2015). Compared to synthetic nanoparticles, EVs exhibit low immunogenicity and cell/tissue tropism due to the surface display of self-antigens and ligands facilitating interaction with specific cell populations (Kamerkar et al., 2017). Furthermore, EVs can traverse biological barriers, such as the blood-brain barrier (BBB), making them promising candidates for delivering cancer therapeutics (Alvarez-Erviti et al., 2011). The loading of small ncRNAs into EVs typically involves two steps: first, EVs are collected from cultured cells, including primary cells, stem cells, and cancer cells, and then purified through centrifugation. Subsequently, small ncRNAs can be loaded into EVs using various methods, such as electroporation, sonication, lipid transfections, freeze-thaw, and pH gradient modulation (Shtam et al., 2013; Usman et al., 2018; Jeyaram et al., 2020; Pottash et al., 2022).

In a recent study, Kamerkar et al. demonstrated that engineered exosomes derived from normal fibroblast-like mesenchymal cells (iExosomes) exhibited prolonged circulation times compared with liposomes. The retention of iExosomes in circulation was found to be correlated with the expression level of CD47 (Kamerkar et al., 2017). In this study, iExosomes were loaded with siRNA or shRNA targeting *KRAS* ^*G12D*^ using an optimized electroporation technique. The silencing of *KRAS* ^*G12D*^ in the cancer cells mediated by iExosomes relied on the enhanced macropinocytosis triggered by oncogenic *RAS*. Repeated intraperitoneal injection of iExosomes effectively suppressed *KRAS* ^*G12D*^ orthotopic pancreatic tumors and significantly increased overall survival in the PANC-1 tumor-bearing mouse model. A phase I trial is currently underway to assess the potential of iExosomes in patients with metastatic pancreatic ductal adenocarcinoma (PDAC) carrying *KRAS* ^*G12D*^ mutation (NCT03608631).

The development of EV-based small ncRNA therapeutics faces several challenges, including limited production yield, low RNA loading efficiency, and substantial batch-to-batch variation. The production of clinical-grade exosomes often requires large-scale cell culture in bioreactors (Mendt et al., 2018). Additionally, purification via centrifugation requires access to specialized large instruments. There have been extensive efforts to enhance EV production, including physical or chemical stimulation of cultured cells with heat, radiation, or calcium ionophore, mechanical generation of small vesicles through sonication or extrusion, and genetic modification of cells to boost exosome production (Kojima et al., 2018; Usman et al., 2018; Jabbari et al., 2019; Wen et al., 2022).

### 3.4 Viral vectors and bacterial minicells

Various types of viral vectors, including adenoviruses, adeno-associated viruses (AAV), lentiviruses, and retroviruses, have been harnessed for delivering small ncRNAs, including shRNA, pre-miRNA and circular miRNA-sponge, etc. (Herrera-Carrillo et al., 2017). Genetically engineered viral vectors can efficiently deliver transgenes to both dividing and non-dividing cells. Furthermore, viral vectors possess the ability to self-replicate and induce sustained transgene expression either through integration into the host genome or stable extrachromosomal expression. Utilizing viral vectors for small ncRNA delivery eliminates the need for repeated administrations. Kota et al. designed a self-complementary AAV (scAAV) vector by cloning *miR-26a* into the intron of EF1α promoter, co-expressed with eGFP, to treat *MYC*-induced liver tumor. *miR-26a* induces cell cycle arrest at G1 stage in liver cancer cells by downregulating the expression of cyclins D2 and E2. Their study demonstrated that a single intravenous injection of scAAV.miR26a.eGFP induced tumor-specific apoptosis and significantly suppressed tumor growth without inducing toxicity (Kota et al., 2009). However, clinical translation of viral vectors faces several challenges. Integration of the transgene into the host genome can potentially induce oncogenic mutations. Pre-existing neutralizing antibodies in patients can rapidly inactivate viral vectors. Additionally, like EVs, large-scale production of viral vectors is resource-intensive and time-consuming. To overcome these challenges while retaining the benefits of viral vectors, viral-like particles (VLPs) have been developed (Shao et al., 2012).

Bacterium-derived minicells, i.e., small cells without chromosomes, are versatile carriers for cancer therapeutics (MacDiarmid et al., 2007; Jivrajani and Nivsarkar, 2016; Yu et al., 2021). These minicells are generated through ectopic septation of genetically modified Gram-positive or Gram-negative bacteria (MacDiarmid et al., 2007). Notably, siRNAs can traverse the intact membrane of minicells, enabling efficient loading through co-incubation (MacDiarmid et al., 2009). For tumor targeting, the minicell surfaces are frequently modified with bispecific antibodies. These antibodies feature one arm binding to the O-polysaccharide component of the minicell surface lipopolysaccharide, while the other arm recognizes a specific surface marker on cancer cells. An illustrative example is the development of an *EGFR*-targeted bacterium minicell loaded with *miR-16* mimics, known as TargomiRs, engineered for the treatment of malignant pleural mesothelioma (MPM) (Reid et al., 2013). In a murine xenograft model, intravenous injection of TargomiRs effectively inhibited tumor growth in a dose-dependent manner. A dose-escalation Phase I study demonstrated that TargomiRs had an acceptable safety profile and showed signs of effectiveness, paving the way for further clinical investigations (van Zandwijk et al., 2017).

## 4 Small ncRNAs and cancer immunotherapy

In recent years, cancer immunotherapy has emerged as a groundbreaking advancement in cancer treatment. In contrast to conventional chemotherapy and radiation therapy, immunotherapies, such as CAR-T therapy and immune checkpoint inhibition (ICI), provide unprecedented therapeutic benefits across various cancers. The success of these treatments fuels ongoing exploration into additional cancer-associated immunological pathways and novel immune engineering approaches to combat cancer. Many preclinical and clinical studies have elucidated the extensive involvement of ncRNAs in immune suppression within the tumor. Notably, circulating exosomal miRNAs such as *miR-146a*, *miR-125a*, *miR-155*, *let-7e*, *miR-146b*, *miR-125b*, *miR-99b*, and *miR-100* have been identified as markers for immune resistance in melanoma patients undergoing nivolumab and ipilimumab treatment (Huber et al., 2018). Furthermore, miRNAs targeting immune suppressors are often downregulated in the tumor, contributing to an immune-suppressive tumor microenvironment.

The utilization of ncRNAs in immunotherapy is an appealing strategy due to their capacity to regulate the expression of nearly all proteins through translational repression, unlike blocking antibodies that can only target cell surface ligands. The rational design and swift turnaround time of ncRNAs facilitate the rapid assessment of therapeutic strategies. Moreover, ncRNA therapeutics targeting immunological pathways can leverage existing delivery platforms for cancer treatment, with the caveat that the delivery target must be tailored for specific subsets of immune cells or cancer cells. It is noteworthy that the small size and similar chemistry of ncRNAs makes them particularly suitable for combination therapy, wherein two or more ncRNAs targeting complimentary pathways can be synchronized for more robust anti-tumor immune responses.

Cancer-associated immune responses involve the intricate interplay among effectors cells, antigen-presenting cells, stromal cells, and cancer cells within the tumor microenvironment (Chen and Mellman, 2013). Solid tumors evade immune surveillance by reducing antigen presentation, minimizing T-cell infiltration, and upregulating the inhibitory signals that induce T-cell exhaustion. ncRNAs are commonly utilized to disrupt inhibitory pathways, thereby enhancing the efficacy and duration of immune responses. Early studies targeted T-cells directly with siRNAs to attenuate their response to inhibitory signals, such as siRNAs targeting *CD25* or *Smad4* to dampen the T-cell response to IL2 or TGF-β, respectively (Rajagopalan et al., 2017; Puplampu-Dove et al., 2018). These siRNAs were complexed with an oligonucleotide (ODN) aptamer binding to 4-1BB, a ligand transiently expressed by activated T cells. Similarly, Shobaki et al. developed LNPs loaded with siRNAs targeting the signal transducer and activator of transcription 3 (STAT3) and hypoxia-inducible factor 1α (HIF-1α), promoting macrophage infiltration and polarization to a proinflammatory M1 phenotype in a xenografted human renal cell carcinoma model (Shobaki et al., 2020).

Programmed cell death protein 1 (PD1) and its ligand, PDL1, are crucial immune checkpoints in solid tumors. PDL1 expression on cancer cells or myeloid-derived suppressor cells (MDSCs) allows binding to PD1 on activated T-cells, leading to the inhibition of their activation and cytokine production. Despite the notable success of monoclonal antibodies targeting the PD1/PDL1 axis in clinical applications, resistance to PD1/PDL1 inhibition alone is observed in many cancer types (Mahoney et al., 2015). Consequently, several studies have undertaken a strategy of combining siRNAs against *PDL1* with complimentary immune modulators to elicit robust immune responses. Analogous to the previously mentioned ncRNA cancer therapeutics, these siRNAs are frequently formulated with cationic polymers or nanoparticles and conjugated with targeting ligands for effective tumor homing and endosomal escape. For instance, in a mouse model of ovarian cancer, Teo et al. demonstrated that siRNA complexed with folate-modified polyethyleneimine (PEI) sensitized cancer cells to adoptive T cell therapy (Teo et al., 2015). Similarly, Zhang et al. showcased targeted delivery of siRNAs using a lipid vesicle containing a cationic core of protamine (Zhang et al., 2022). Co-delivery of siRNA against *Pdl1* with a TGF-β inhibitor efficiently inhibited tumor progression in a mouse model of triple negative breast cancer. More recently, Liu et al. developed a nanovaccine using a cholesterol-modified antimicrobial peptide combined with three siRNAs against *Stat3*, *Ccr2* and *Tgf-β* (Liu et al., 2023). Intratumoral injection of the nanovaccine increased the immune response to anti-PD1 therapy in a cold mouse B16F10 melanoma model. These studies highlight the potential of ncRNAs in cancer immunotherapy, offer both potent therapeutic targets and versatile strategies for immunomodulation.

## 5 Discussion

Over the past decade, small ncRNAs have emerged as promising cancer therapeutics due to their remarkable ability of translational regulation. In line with Paul Ehrlich’s “magic bullet” concept, the therapeutic effectiveness of small ncRNA drugs is heavily contingent on their precise delivery to cancer cells, while sparing normal cells from adverse effects. Major advancements in chemical modifications and LNP formulations have culminated in several FDA approvals for small ncRNA therapeutics, with the majority of successes observed in treating liver-related diseases. However, achieving specific and efficient delivery to cancer cells in other organs remains a formidable obstacle in the clinical translation of small ncRNA-based cancer treatments. Indeed, suboptimal delivery efficiency has been the primary cause of therapeutic ineffectiveness, leading to the termination of several clinical trials.

On a more optimistic note, ongoing clinical studies have yielded invaluable insights into potential solutions. Firstly, the judicious selection of disease targets based on the unique properties of small ncRNA formulations is crucial. Targeting locally advanced tumors with unmodified small ncRNAs can bypass many transport barriers and holds promise for imminent clinical success. Secondly, achieving efficient systemic delivery necessitates the precise modulation of delivery vehicle properties at various stages of *in vivo* transport. Biogenic particles, such as exosomes, viruses, and bacterium-derived carriers, honed through millions of years of evolution, exhibit enhanced potential for systemic small ncRNA therapeutic delivery. In this context, we anticipate a burgeoning field of biomimetic delivery vehicles, capable of fully harnessing the therapeutic potential of small ncRNA therapeutics.
